# Ca^2+/^Calmodulin-dependent kinase II delta B is essential for cardiomyocyte hypertrophy and complement gene expression after LPS and HSP60 stimulation *in vitro*

**DOI:** 10.1590/1414-431X20198732

**Published:** 2019-07-15

**Authors:** C.V. Cruz Junho, M. Trentin-Sonoda, J.M. Alvim, F. Gaisler-Silva, M.S. Carneiro-Ramos

**Affiliations:** 1 Centro de Ciências Naturais e Humanas, Universidade Federal do ABC, Santo André, SP, Brasil; 2 Cellular and Molecular Medicine, Faculty of Medicine, University of Ottawa, Ottawa, Ontario, Canada; 3 Laboratorio de Genética e Cardiologia Molecular, Instituto do Coração, Hospital das Clínicas, Faculdade de Medicina, Universidade de São Paulo, São Paulo, SP, Brasil

**Keywords:** Cardiomyocytes, Hypertrophy, Inflammation, Complement, TLR 2/4, CaMKII

## Abstract

Inflammation plays an important role in the development of cardiovascular diseases (CVDs), suggesting that the immune system is a target of therapeutic interventions used for treating CVDs. This study evaluated mechanisms underlying inflammatory response and cardiomyocyte hypertrophy associated with bacterial lipopolysaccharide (LPS)- or heat shock protein 60 (HSP60)-induced Toll-like receptor (TLR) stimulation and the effect of a small interfering RNA (siRNA) against Ca^2+^/calmodulin-dependent kinase II delta B (CaMKIIδB) on these outcomes. Our results showed that treatment with HSP60 or LPS (TLR agonists) induced cardiomyocyte hypertrophy and complement system C3 and factor B gene expression. *In vitro* silencing of CaMKIIδB prevented complement gene transcription and cardiomyocyte hypertrophy associated with TLR 2/4 activation but did not prevent the increase in interleukin-6 and tumor necrosis factor-alfa gene expression in primary cultured cardiomyocytes. Moreover, CaMKIIδB silencing attenuated nuclear factor-kappa B expression. These findings supported the hypothesis that CaMKIIδB acts as a link between inflammation and cardiac hypertrophy. Furthermore, the present study is the first to show that extracellular HSP60 activated complement gene expression through CaMKIIδB. Our results indicated that a stress stimulus induced by LPS or HSP60 treatment promoted cardiomyocyte hypertrophy and initiated an inflammatory response through the complement system. However, CaMKIIδB silencing prevented the cardiomyocyte hypertrophy independent of inflammatory response induced by LPS or HSP60 treatment.

## Introduction

Cardiac hypertrophy (CH) is a compensatory response in the heart induced by various physiological or pathological stimuli. Hypertrophy is essential for increasing contraction strength during response to pressure overload. However, if the induced hypertrophic response is not ceased after the cessation of the physiological or pathological stimuli, it can lead to the development of cardiovascular diseases (CVDs) and eventually heart failure (1,2). Some proinflammatory cytokines play an important role in the development of CH (3,4). Moreover, studies have reported the involvement of both Toll-like receptors (TLRs) and complement system (5) in the development of CH. TLR2 and TLR4 are the most abundant TLRs in the heart tissue and the most studied TLRs in this field (6). TLR4 recognizes pathogen-associated molecular patterns (PAMPs) such as bacterial lipopolysaccharide (LPS), and endogenous molecules called damage-associated molecular patterns (DAMPs) such as heat shock proteins (HSPs), HMGB1, fibronectin, etc., which are released during a stress response (7).

The complement system forms the main component of the humoral inflammatory response along with antibodies (8). The classical pathway of humoral inflammation is activated by IgM or IgG antibodies that form complexes with antigens (9). Lectin pathway of the complement system depends on the recognition of carbohydrate residues present on cell membranes. An alternative pathway of the complement system is activated by the self-hydrolysis of complement C3, which promotes the binding of the b subunit of C3 to the b subunit of complement factor B (CFB) to produce a membrane attack complex (9). Although the complement system often induces an inflammatory response against pathogenic invasion, it may also induce an inflammatory response by recognizing endogenous factors, including inflammation in the cardiac tissue. Receptors of the complement system interact with TLRs to induce an inflammatory response by inducing inflammatory gene transcription through the nuclear translocation of nuclear factor-kappa B (NF-κB). Despite the importance of the complement system in CVDs, only a few studies have examined the role of the complement system in CVDs. In 2009, Singh et al. (10) reported that Ca^2+^/calmodulin-dependent kinase II (CaMKII)-induced activation of NF-κB pathway triggered CFB expression in a mouse model of myocardial infarction, suggesting that CaMKII functions as a link between inflammation and CH (10,11).

In the present study, we hypothesized that TLR stimulation by a DAMP such as HSP60 induced an inflammatory response and cardiomyocyte hypertrophy, and that these outcomes were abrogated after treatment with a small interfering RNA (siRNA) against CaMKII delta B (CaMKIIδB). Here, we found that TLR stimulation by HSP60 was comparable to that by LPS and was sufficient to induce cardiomyocyte hypertrophy and C3 and CFB expression. Moreover, we observed that CaMKIIδB silencing by using the specific siRNA prevented the increase in inflammatory mediator expression, cardiomyocyte hypertrophy, and NF-κB expression.

## Material and Methods

### Primary culture of cardiomyocytes

The present study was performed according to the Brazilian Federal Law No. 11,794 of 2008, which regulates the use of animals in scientific experiments, and according to protocol No. 7400050716 approved by the Research Ethics Committee of the Federal University of ABC.

Cardiomyocytes were cultured using a protocol described previously by Barreto-Chaves et al. (12). Cardiomyocytes were obtained from neonate Wistar rats (1 to 3 days old). For cardiomyocyte collection, the animals were disinfected with 70% ethanol and euthanized through decapitation. The sternums of the rats were cut to expose the hearts. The hearts were rapidly harvested using tweezers and were placed in a Petri dish containing 1× ADS (NaCl, HEPES, NaH_2_PO_4_, d-glucose, KCl, and MgSO_4_). The atria, connective tissue, and fat remnants were removed, and only the ventricles were cut and transferred to a conical tube containing 6 mL pancreatin-based digestion buffer and collagenase (Sigma-Aldrich/Worthington Biochemical Corporation, USA). The ventricles were incubated at 37°C with agitation at 1200 *g* for 30 min, and the supernatant obtained was discarded. Next, 6 mL digestion buffer was added to the tube, and the ventricles were incubated further under the same conditions for 30 min. Supernatant obtained was aspirated, transferred to another conical tube containing 1 mL newborn calf serum (NCS; Gibco, Invitrogen, USA), and centrifuged at 1200 *g* for 5 min, at 25°C. The cell pellet formed was resuspended in 1.5 mL NCS, placed in a Petri dish, and maintained in a CO_2_ oven until further use. Enzymatic digestion was repeated approximately 5–6 times until small whitish pieces of the ventricles were obtained. The cells isolated after each enzymatic digestion were centrifuged at 1200 *g* for 5 min, at 25°C. The cell pellet obtained was resuspended in 1.5 mL NCS and centrifuged at 3000 *g* for 30 min at 25°C using a Percoll density gradient medium (GE Healthcare, USA), which separates cardiomyocytes from the other cell types present in the heart.

Cardiomyocytes obtained in this step were placed in six-well plates (Gibco, Invitrogen) containing 5% NCS, 10% horse serum (HS), and 1% streptomycin and penicillin, and the plates were incubated under sterile conditions in a heater with an atmosphere of 5% CO_2_, temperature of 37°C, and humidity of 80%. The cells were treated as described below. Before administering the treatments, the cells were cultured in a serum-starved medium for 16 h, after which the culture medium was replaced with Dulbecco's modified Eagle's medium (DMEM; Gibco) containing 0.5% NCS. Next, the cells were treated with 0.01, 0.1, 1, 10, or 100 μM *Escherichia coli* LPS (13) or 1, 5, or 10 μM HSP60 (14). The response of cardiomyocytes to the TLR 2/4 agonists was analyzed by dividing the cells into the following groups: control group (cells cultured in DMEM), LPS group (cells treated with LPS for 24 h), and HSP60 group (cells treated with HSP60 for 24 h).

### Evaluation of cardiomyocyte hypertrophy

Cardiomyocyte hypertrophy induced by the TLR 2/4 agonists was measured by calculating cardiomyocyte surface area. For this, 50 cardiomyocytes that were randomly obtained from different fields were examined under a light microscope at 200× magnification. Images of the examined cardiomyocytes were digitized, and their surface area was measured using ImageJ software (NCBI, NIH, USA). Expression of the α-actin and BNP genes (CH biomarkers) was determined by performing real-time polymerase chain reaction (real-time PCR) (detailed below).

### siRNA assay

The involvement of CaMKIIδB in TLR 2/4-induced CH was evaluated using a siRNA targeting the sequence 5′-GUUCGAGUGUUCAGAUGAUdTdT-3′ of CaMKIIδB. Transfection efficiency was evaluated using a non-specific siRNA sequence conjugated with a fluorescent molecule (BLOCK-IT™ Fluorescent Oligo; Invitrogen, USA). Cardiomyocytes were transfected with different doses of the siRNAs to determine the dose that provided the highest degree of gene silencing and consequently decreased the corresponding protein expression.

siRNA transfection protocol used in the present study was the same as that described by Diniz et al. (15). Cardiomyocytes were cultured in the primary culture, as described above. Next, the cells were plated at a density of 1.9×10^5^ cells/well in a six-well plate and maintained at 37°C and 80% humidity in a 5% CO_2_ incubator under sterile conditions. The cells were examined each day under a microscope to determine their typical morphology and spontaneous contractility, which are the indicators of cell viability. In addition, the culture medium was replaced every 24 h until the time of the treatment. At 24 h after extraction by performing the Percoll density gradient centrifugation, the cardiomyocytes were seeded in a serum- and antibiotic-free DMEM for 2 h, followed by transfection with the indicated siRNAs. Next, the cells were divided into the previously defined groups.

After incubation in the transfection medium (containing the indicated siRNAs and Lipofectamine, Invitrogen, USA) for 6 h at 37°C and 80% humidity in a 5% CO_2_ incubator under sterile conditions, the culture medium was replaced again with DMEM containing 5% NCS and 10% HS but no streptomycin and penicillin. At 24 or 48 h after the transfection, the cells were analyzed under a microscope to verify their confluence, morphology, and contractility, which are the markers of cell viability. Positive control cells were analyzed under a fluorescence microscope (Olympus IX71, Japan) with an FITC filter to evaluate transfection efficiency of the CaMKIIδB siRNA. Gene silencing was confirmed by extracting total proteins from the cells and by analyzing CaMKIIδB expression performing western blotting analysis.

The cells were divided into the following experimental groups: control group (cells cultured in DMEM alone), positive control group (cells transfected with 100 ηM siRNA conjugated with the BLOCK-iT™ Fluorescent Oligo), negative control group (cells transfected with 100 ηM siRNA), CaMKIIδB siRNA + LPS group (cells transfected with 100 ηM CaMKIIδB siRNA and treated with 100 μM LPS), and CaMKIIδB siRNA + HSP60 group (cells transfected with 100 ηM CaMKIIδB siRNA and treated with 1 μM HSP60).

### Determination of cell viability and purity

Initial cell viability was analyzed using trypan blue reagent before plating a known number of cells (4×10^4^ cells/cm^2^) in each well. The cells were cultured, and the culture medium was replaced once daily in the same manner as that described in the treatment experiments. Control cells were cultured in a similar manner but were not administered the indicated treatments. After incubation for a stipulated time period, the medium present in each well was transferred to a 2-mL conical tube. The cells were then washed with 1× phosphate-buffered saline (PBS) to remove serum residue present in the wells, and this PBS was collected in new tubes. Next, 200 μL 0.25% trypsin (Sigma, USA) was added to each well, and the plate was incubated at 37°C for 6 min to allow the enzyme to release the cells from the plate. Each well was analyzed under a microscope to confirm the release of all the cells from the plate. Next, 500 μL cell culture medium that was collected initially was placed in the corresponding wells to stop the enzyme activity. Next, all the contents of each well were collected, transferred to conical tubes, and centrifuged at 10,000 *g* for 15 min at 37°C.

Supernatant obtained was discarded, and the cell pellet formed was resuspended in 500 μL culture medium. Next, 10 μL cell suspension was mixed with 10 μL trypan blue (Sigma), and cells were counted under a microscope in a Neubauer chamber. The cells were counted in duplicate, and the mean was used for analyzing the viability of cells treated with the TLR 2/4 agonists and transfected with the different siRNA doses for different time periods. Results are reported as percentage (16).

The purity of cardiomyocytes extracted using the Percoll density gradient centrifugation was determined by phenotypically characterizing the cells by immunocytochemical analysis with antibodies against sarcomeric tropomyosin (Sigma, USA), which is a cardiomyocyte marker, and vimentin (Sigma, USA), which is a fibroblast marker, as negative control. A specific fluorescence molecule-conjugated secondary antibody was used for detecting the primary antibody. The slides were fixed and stained with ProLong^®^ Gold Antifade Mountant and DAPI (Thermo Fisher Scientific, USA), which stained the nuclei in blue, and were analyzed under a confocal microscope (DMI 6000; Leica, German).

### Gene expression and protein content analyses

Total RNA was extracted from cardiomyocytes using Trizol, followed by treatment with isopropanol for RNA precipitation. The pellets obtained were washed with an aqueous solution of 75% ethanol. RNA concentration was measured using NanoDrop Lite spectrophotometer (Thermo Fisher Scientific). The isolated RNA was reverse transcribed using ProtoScript kit (New England BioLabs, USA), and the obtained cDNA was used to perform real-time PCR (Stratagene^®^ Mx3005P; Agilent Technologies, USA) to quantify gene expression. The mRNA levels of the α-actin, C3, CFB, NF-κB (p65 subunit), and cyclophilin A (housekeeping gene) genes were measured using primers listed in Table 1.

**Table 1:** List of primers.

Target Gene	Sense	Anti-sense
Cyclophilin A	5′-AGCATACAGGTCCTGGCATC-3′	5′-AGCTGTCCACAGTCGGAAAT-3′
α-actin	5′-GGCAAGATGAGAGTGCACAA-3′	5′-CGGAGAATGATGGTCCAGAT-3′
BNP	5′-AAGCTGCTGGAGCTGATAAGA-3′	5′-GTTACAGCCCAAACGACTGA-3′
C3	5′-GACTGGGATGTCACCCTGAG-3′	5′-TCACTATGGGACCAGCTTCA-3′
CFB	5′-CTTCCTCTTCTGCTGTTCTCC-3′	5′-AGCCTTCCTGCCAAGATTCC-3′
CFH	5′-GCCAAGTGTTCGGTATCCAG-3′	5′-GGAGTCAGTTGGTCCCAGAA-3′
NF-kB	5′-ATGGCAGACGATGATCCCTAC-3′	5′-CGGAATCGAAATCCCCTCTGTT-3′
IL-6	5′-TAGCCGCCCCACACAGACAG-3′	5′-GGCTGGCATTTGTGGTTGGG-3′
TNF-α	5′-GCCTCTTCTCATTCCTGCTTG-3′	5′-CTGATGAGAGGGAGGCCATT-3′

Total protein was extracted from cardiomyocytes by lysing the cells with RIPA buffer (0.1 M KCl, 10 mM HEPES, 3 mM MgCl_2_, 5 mM EDTA, 10% glycerol, 1 mM DTT, 10% SDS, and 0.1 mL proteinase inhibitor cocktail). Protein content was quantified using BCA kit (Thermo Fisher Scientific) on a 96-well plate using a plate reader. Next, equal amounts of the extracted proteins were loaded in each well of a 12% polyacrylamide gel and were electrophoresed for 90 min at a constant voltage of 125 V. Next, the proteins resolved on the gel were blotted onto a nitrocellulose membrane through electrophoretic transfer at a constant voltage of 90 V for 1 h. The membrane was washed and probed overnight at 4°C with primary antibodies against β-actin (Immunity Inc., Brazil) and total CaMKII (Genetex, USA). After washing, the membrane was incubated with corresponding peroxidase-conjugated secondary antibodies for 1 h at room temperature. Finally, the membrane was analyzed using ECL system (Thermo Fisher Scientific). Protein bands were visualized using ChemiDoc^®^ (BioRad, USA), and band densities were measured using ImageJ software. The antibodies used in the present study are listed in Table 2.

**Table 2:** List of antibodies.

Target protein	Molecular weight (kDa)	Type	Dilution	Source
CaMKIIδ	56	Rabbit polyclonal	1:500	Genetex, GTX111401
α-actin	42	Rabbit polyclonal	1:500	Imuny, IM-0075
Rabbit IgG	Secondary	Goat polyclonal	1:5000	Jackson Immuno Research, #011-000-003

### Statistical analysis

Statistical analyses were performed using GraphPad Prism 6 (USA). Data are reported as means±SE. Groups were compared using one-way analysis of variance followed by Bonferroni post-test or *t*-test, as indicated in figure legends, and P<0.05 was considered to be statistically significant.

## Results

### LPS and HSP60 induced cardiomyocyte hypertrophy and complement gene expression

**Figure 1.**
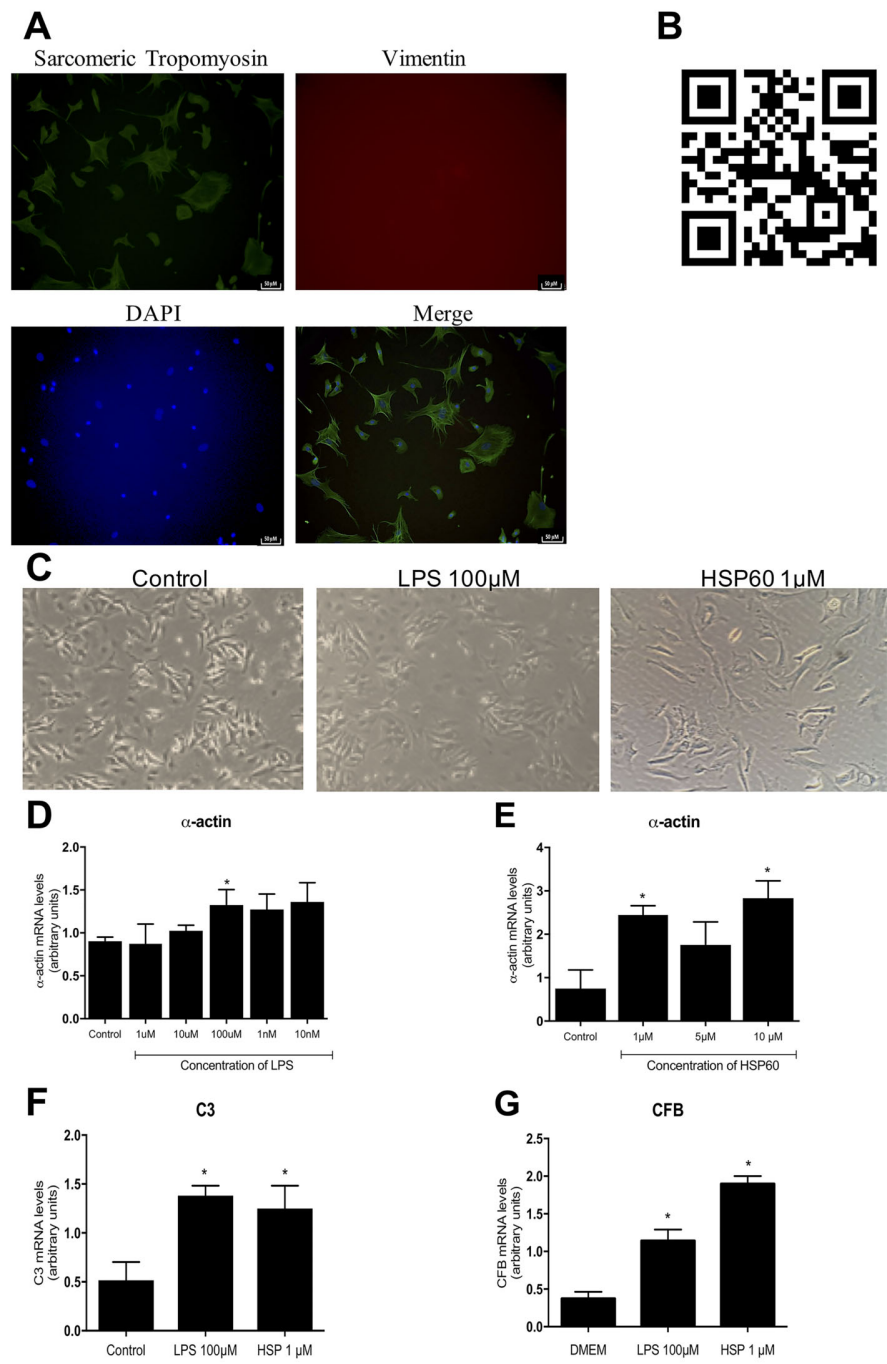
Effect of Toll-like receptor (TLR) ligands on complement system expression *in vitro.* **A**, Representative image of primary cultured of cardiomyocytes in immunocytochemistry for sarcomeric tropomyosin and for vimentin by a confocal microscope. The nucleus was stained with blue DAPI and then the images were merged (200×, scale bar 50 μm). **B**, Link to a representative video of the primary culture of cardiomyocytes after 24 h of extraction. **C**, Representative image of the primary culture of cardiomyocytes before treatments (control), after treatment of 100 μM of lipopolysaccharides from *E.Coli* (LPS), and after treatment of 1 μM of heatshock protein 60 (HSP60) (100×). **D**, Analysis of α-actin (cardiac hypertrophy marker) gene expression by real time PCR in cardiomyocytes treated with different concentrations of LPS for 24 h, and **E**, treated with different concentrations of HSP60 for 24h (n=4 in all groups). **F**, Analysis of the gene expression of C3, and **G**, of CFB evaluated by real time PCR in cardiomyocytes treated with HSP60 and with LPS for 24 h (n=4). Data reported as means± SE. *P<0.05 *vs* the corresponding Control group (ANOVA).

Cardiomyocytes were cultured successfully, as shown in Figure 1. The absence of vimentin expression was determined by immunocytochemical analysis and indicated the absence of fibroblast contamination, thus confirming the efficacy of isolating cardiomyocytes by performing Percoll density gradient centrifugation (Figure 1A). Cardiomyocyte contractility can be observed using a QR code technology, which provides access to a video of cells in culture (Figure 1B). The cells were treated with the TLR 2/4 agonists LPS and HSP60, and dose-response curves were obtained. We observed that treatment with 100 μM LPS increased α-actin expression compared with that in control cells (P<0.05, Figure 1D). Similarly, treatment with 1 μM HSP60 increased the expression of the α-actin gene, a well-established molecular marker of cardiomyocyte hypertrophy, compared with that in control cells (P<0.05, Figure 1E). These results validate the efficiency of the two treatments in inducing CH. To reinforce the result related to cardiomyocyte hypertrophy, we calculated cardiomyocyte surface area to confirm the effect of the siRNA-induced silencing of CaMKIIδB (see Figure 3A).

After confirming that the TLR 2/4 agonists induced cardiomyocyte hypertrophy, we determined mechanisms underlying this process. For this, we examined the role of the complement system *in vitro* by evaluating the expression of complement C3 and CFB genes in cardiomyocytes treated with 1 μM HSP60 or 100 μM LPS. We observed that the expression of both genes increased in cardiomyocytes treated with LPS or HSP60 (Figure 1F and G).

### CaMKIIδB silencing prevented TLR 2/4 agonist-induced CH

**Figure 2.**
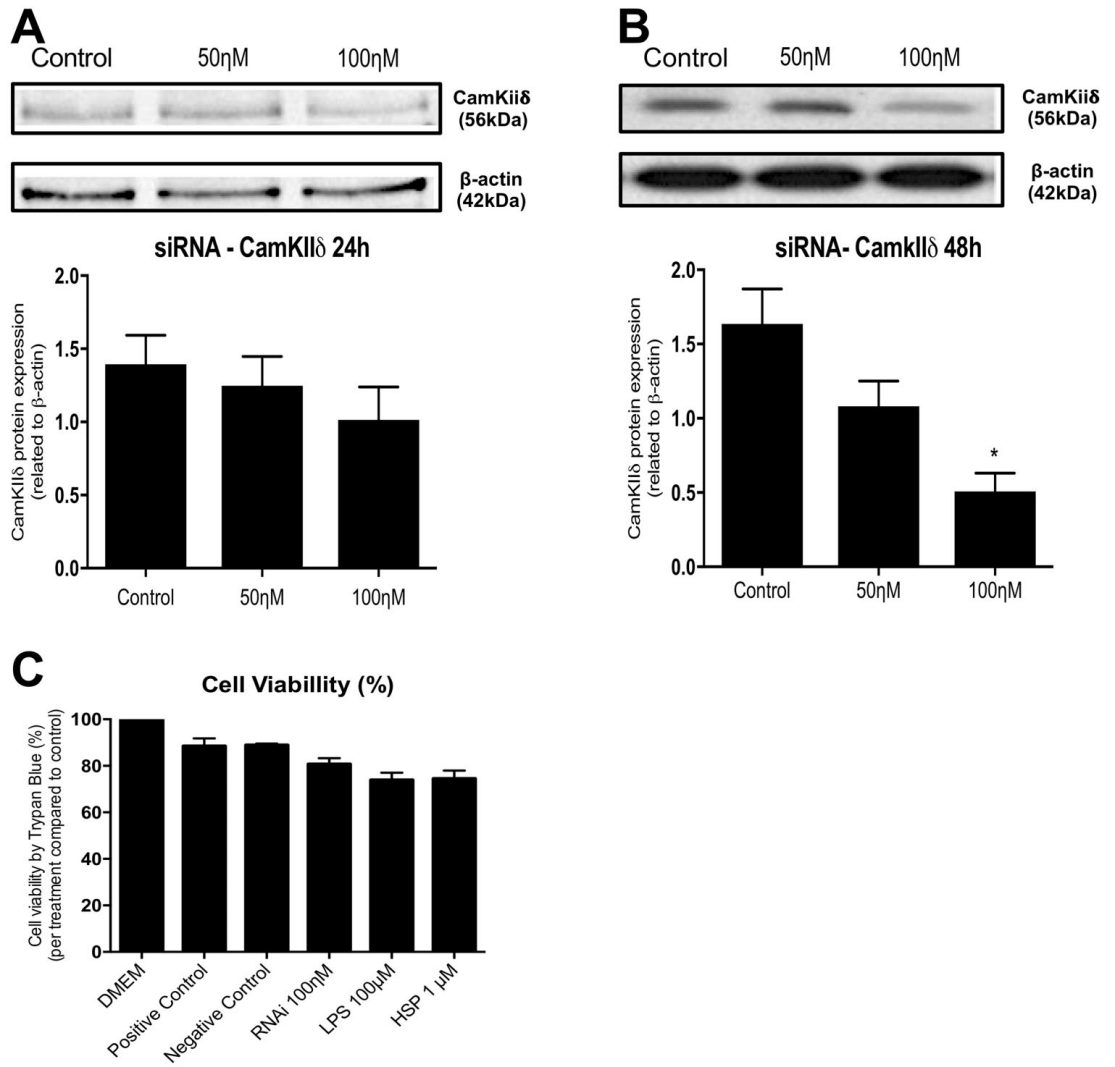
Silencing of CamKII and its effects on cellular viability. **A**, Representative images of western-blotting and protein expression of the silenced protein CamKIIδ in cardiomyocytes treated with 0.05 and 0.1 μM of siRNA for 24 h and **B**, for 48 h. **C**, Cellular viability after treatments compared to control/RNAi control cells. Data are reported as means±SE. *P<0.05 *vs* Control (n=4 in all experimental groups; ANOVA).

The transfection efficiency of the siRNA against CaMKIIδB was evaluated by transfecting cultured cardiomyocytes with two different doses of the siRNA, namely, 50 ηM and 100 ηM, for 24 and 48 h. We observed that treatment with 100 ηM CaMKIIδB siRNA for 48 h significantly decreased CaMKIIδB protein expression compared with treatment with 100 ηM CaMKIIδB siRNA for 24 h (P<0.05; Figure 2A and B), indicating that treatment with 100 ηM CaMKIIδB siRNA for 48 h was the most effective for silencing CaMKIIδB expression. However, treatment with 100 ηM CaMKIIδB siRNA for 48 h produced a faint protein band during western blotting analysis. This may be because western blotting analysis to detect CaMKIIδB expression was performed using an antibody against total CaMKII and may have detected CaMKII delta C (CaMKIIδC) (cytoplasmic isoform).

At 24 h after obtaining the cardiomyocytes by performing Percoll density gradient centrifugation, the cardiomyocytes were treated with the CaMKIIδB siRNA for 48 h, followed by treatment with the TLR 2/4 agonists (100 μM LPS or 1 μM HSP60). Figure 2C shows that all the experimental conditions used in this study did not decrease the viability of the extracted cardiomyocytes.

**Figure 3.**
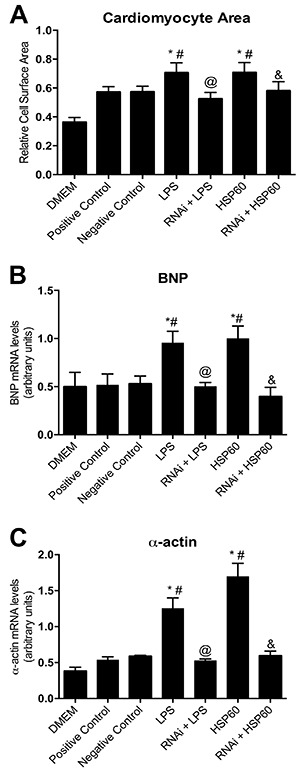
Silencing of CamKIIδ and its effects on cardiac hypertrophy. **A**, Relative cell surface demonstrating the cardiomyocyte area after treatments compared to control/RNAi control cells. **B**, Analysis of BNP (brain natriuretic peptide, cardiac hypertrophy marker) and **C**, α-actin (cardiac hypertrophy marker) evaluated by real time PCR in cardiomyocytes treated with 0.1 μM of the interference RNA for 48 h, followed by 100 μM of LPS or 1 μM of HSP60 for 24 h and control cells. Data are reported as means±SE. *P<0.05 *vs* DMEM, ^#^P<0.05 *vs* RNAi positive control, ^@^P<0.05 *vs* LPS treatment, ^&^P<0.05 *vs* HSP60 treatment (n=4 in all experimental groups, ANOVA).

We observed that CH was significantly attenuated in cardiomyocytes transfected with the CaMKIIδB siRNA and treated with the TLR 2/4 agonists (P<0.05). The increase in cardiomyocyte surface area induced by LPS or HSP60 treatment (P<0.05) decreased in cardiomyocytes pretreated with the CaMKIIδB siRNA, suggesting that CaMKIIδB silencing prevented cardiomyocyte hypertrophy (Figure 3A). Moreover, the expression of CH markers, namely, brain natriuretic peptide (BNP) and α-actin, significantly decreased in CaMKIIδB-silenced cardiomyocytes (P<0.05) compared with that in control and LPS- or HSP60-treated cardiomyocytes (Figure 3B and C). BNP and α-actin expression levels in LPS- or HSP60-treated cardiomyocytes pretreated with the CaMKIIδB siRNA were almost similar to those in untreated cells, indicating that CaMKIIδB-silencing prevented cardiomyocyte hypertrophy. These results highlight the importance of CaMKIIδB in the development of TLR 2/4-mediated CH.

### CaMKIIδB silencing prevented complement component expression and NF-kB expression

**Figure 4.**
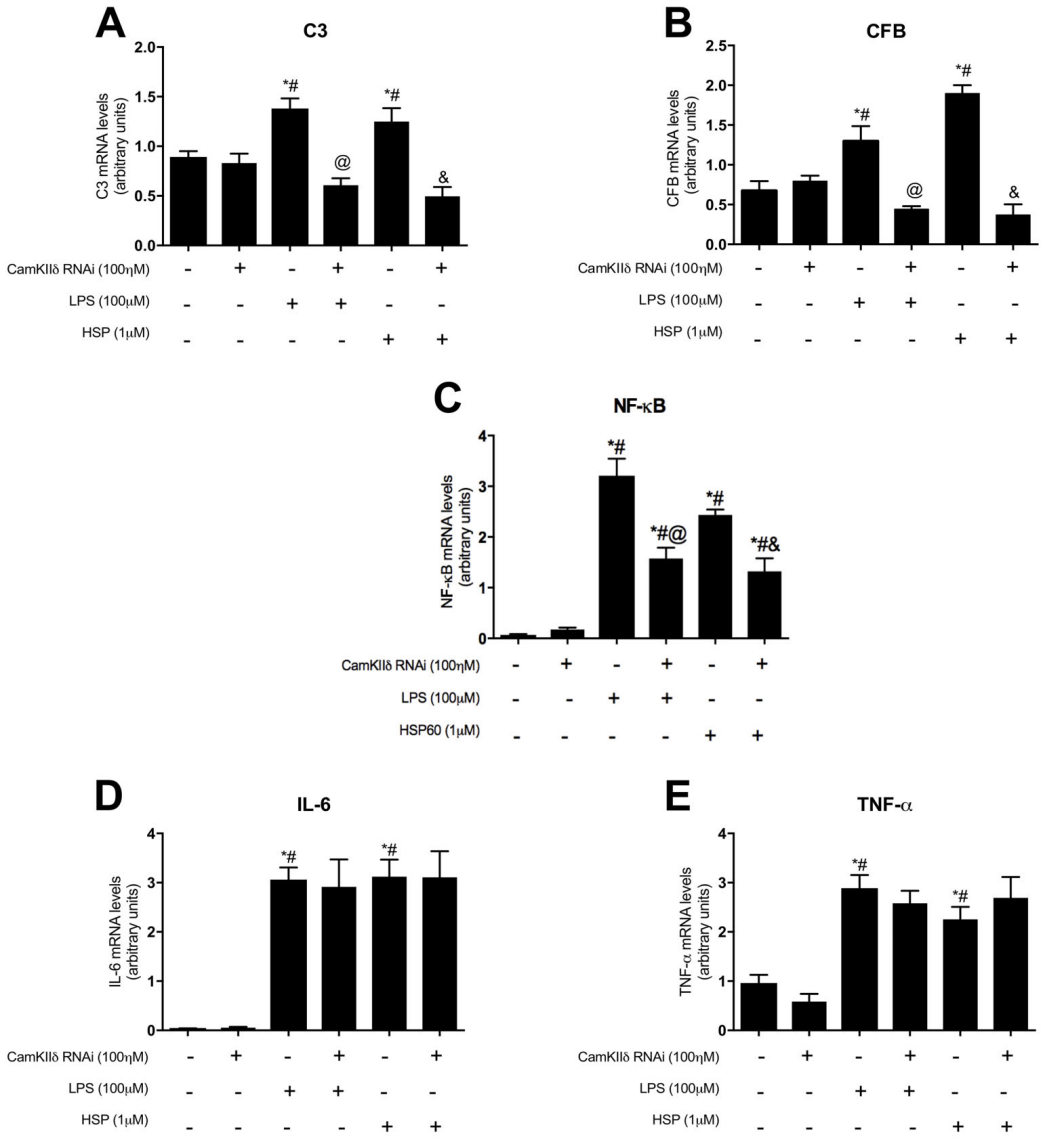
Effects of the silent CaMKIIδ in the complement system components. **A**, Analysis of C3 gene, **B**, CFB gene, **C**, NF-κB, **D**, IL-6, and **E**, TNF-α gene expressions evaluated by real time PCR, in cardiomyocytes treated with 0.1 μM of the interference RNA for 48 h, followed by 100 μM of LPS or 1 μM of HSP60 for 24 h, and control cells. Data are reported as means±SE. *P<0.05 *vs* Control, ^#^P<0.05 *vs* RNAi control; ^@^P<0.05 *vs* LPS treatment; ^&^P<0.05 *vs* HSP60 treatment (ANOVA).

We observed that complement component levels increased significantly in cardiomyocytes treated with the TLR 2/4 agonists (P<0.05) compared with those in control cardiomyocytes. However, pretreatment with the CaMKIIδB siRNA prevented the TLR 2/4 agonist-induced increase in C3 and CFB mRNA expression levels (Figure 4A and 4B). Next, we assessed NF-kB (p65 subunit) gene expression and found that treatment with both of the TLR 2/4 agonists significantly increased NF-kB mRNA level (P<0.05) compared with that in control cells. In contrast, CaMKIIδB silencing attenuated the TLR 2/4 agonist-induced increase in the NF-kB mRNA level; however, the NF-kB mRNA level in the CaMKIIδB siRNA-pretreated cardiomyocytes was still higher than that in untreated cardiomyocytes (P<0.05; Figure 4C).

### CaMKIIδB silencing did not alter inflammatory cytokine expression

We observed that TLR 2/4 agonist treatment significantly increased IL-6 and TNF-α expression (P<0.05) compared with that in control cardiomyocytes (Figure 4D and E). However, no significant change in IL-6 and TNF-α expression was observed in the cardiomyocytes pretreated with the CaMKIIδB siRNA.

## Discussion

Several studies, including our previous study (17,18), have highlighted the importance of inflammation in CVD development. One study reported that TLR4 silencing prevented the increase in NF-kB level and development of CH in a model of induced systemic inflammation (17). Cardiac electrical dysfunction can be mediated by mechanisms involving MyD88 and NF-kB, which function downstream of TLRs (18,19). In the present study, we observed that TLR 2/4 activation by LPS or HSP60 increased CH marker expression. Tian et al. (20) reported that rat neonatal cardiomyocytes treated with HSP60 showed increased cytokine expression. However, the present study is the first to show that HSP60 induced cardiomyocyte hypertrophy.

In the present study, TLR 2/4 stimulation by agonists HSP60 and LPS upregulated C3 and CFB gene expression, suggesting that DAMP and PAMP recognition induces the complement system. This can be explained by the crosstalk between these two components of innate immunity, as indicated by the results of studies involving human and mouse *in vitro* and *in vivo* experimental models (21–23). The crosstalk between the complement system and TLRs may induce inflammation both at the innate and adaptive immunity levels.

Distinct TLRs share the same pathways. Classically, TLR stimulation induces MyD88 adaptor protein to promote signal transduction by activating the IKK complex. Subsequently, the IKK complex phosphorylates IκB, a NF-kB regulatory protein, thus promoting its nuclear translocation. However, recent studies indicate that TLR downstream network is more complex than that assumed previously and involves inflammasome activation or CaMKII participation (10). CaMKII belongs to a protein family that has been intensively studied over the past decades (10). Moreover, the role of CaMKII in heart excitation-contraction coupling has been well described. In the present study, primary cardiomyocytes were pretreated with the CaMKIIδB siRNA, followed by treatment with either of the two TLR 2/4 agonists. CaMKIIδB silencing prevented the development of cardiomyocyte hypertrophy, as indicated by the decrease in α-actin and BNP mRNA levels compared with that in control siRNA-treated and untreated cardiomyocytes. Similarly, CaMKIIδB silencing prevented the upregulation of C3 and CFB gene expression and attenuated NF-kB gene expression. CaMKIIδB regulates both hypertrophy and inflammation, and CaMKIIδB silencing may reduce inflammation, thus attenuating the hypertrophic response (11,24). The results of the present study are consistent with those of study by Singh et al. (10), which reported that LPS-stimulated increase in CFB expression was directly associated with CaMKII-induced activation of the NF-κB pathway both *in vitro* and *in vivo* (10). Another study reported that CFB-knockout spontaneously hypertensive rats show a relative reduction in left ventricle mass and cardiomyocytes compared with wild-type rats (25), indicating that an alternative pathway of the complement system is involved in inducing hypertrophy in response to a stress stimulus.

CaMKIIδC is suggested to act as a link between inflammation and CH by regulating NF-kB. The results of the present study showed that although CaMKIIδB silencing was sufficient to prevent HSP60-induced cardiomyocyte hypertrophy, it did not decrease inflammatory cytokine IL-6 and TNF-α levels. These findings are consistent with those of a 2017 study by Gray et al. (26), which reported that CaMKIIδC-knockout mice did not show altered IL-6 and TNF-α mRNA expression after induction of myocardial infarction. CaMKIIδ misregulation exerts harmful effects on the heart. CaMKIIδ overactivation increases cytoplasmic Ca^2+^ level and induces reactive oxygen species synthesis (27), leading to contractility impairment and heart failure. Selective CaMKII inhibitors exert protective effects on isoproterenol-treated commercial cells by normalizing Ca^2+^ release channels in the cardiac sarcoplasmic reticulum (28). Finally, Willeford et al. (29) showed that CaMKIIδ activated by Ang II generates a proinflammatory signal involving inflammasome and macrophages recruitment contributing to fibrosis, suggesting that treatment of cardiac inflammation through inhibition of CaMKIIδ can be an interesting strategy for preventing cardiovascular diseases. Because both the immune system and CaMKII are involved in several cardiomyopathies, additional clinical studies should be performed to understand the crosstalk between these components.

**Figure 5.**
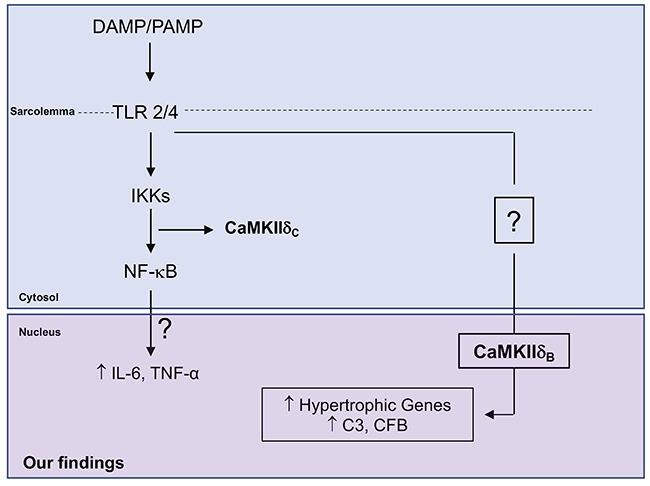
Schematic representation of the study showing the pathways and mechanisms highlighted and their interactions in order to prevent cardiac hypertrophy. DAMP/PAMP: damage-associated molecular patterns/pathogen-associated molecular patterns; TLR: Toll-like receptor; IKK: IKK kinase complex; CaMKIIδB: Ca^2+^/calmodulin-dependent kinase II delta B; NF-κB: nuclear factor-kappa B; IL: interleukin; TNF-α: tumor necrosis factor alpha; C3: complement 3; CFB: complement factor B.

We observed that a stress stimulus induced by HSP60 promoted cardiomyocyte hypertrophy and an inflammatory response, accompanied by an increase of complement system components. Moreover, we observed that CaMKIIδB silencing prevented cardiomyocyte hypertrophy and decreased the inflammatory response. These findings improved our understanding of the mechanisms of CaMKII underlying inflammation-mediated cardiomyopathies (Figure 5).
